# Prognostic value of inflammatory biochemical markers (IL-8, PCT, CRP) for cardiovascular disease in patients with pancreatitis

**DOI:** 10.5937/jomb0-61324

**Published:** 2026-01-28

**Authors:** Zaili Yang, Dejun Cui, Fei Li, Bo Huang, Qi Liu

**Affiliations:** 1 Guizhou Medical University, Graduate School, Guiyang, China; 2 Guizhou Provincial People's Hospital, Department of Gastroenterology, Guiyang, China; 3 Guizhou Medical University, Department of Gastroenterology, Guiyang, China

**Keywords:** pancreatitis, cardiovascular disease, inflammatory biomarkers, clinical biochemistry, prediction model, pankreatitis, kardiovaskularne bolesti, inflamatorni biomarkeri, klinička biohemija, model predikcije

## Abstract

**Background:**

Patients with pancreatitis may be at increased risk of cardiovascular disease (CVD), but the biochemical mechanisms underlying this risk are not fully defined. Inflammatory biomarkers may provide valuable prognostic information.

**Methods:**

We retrospectively analyzed 180 patients with pancreatitis (Jan 2021-Dec 2023). Serum levels of interleukin-8 (IL-8), procalcitonin (PCT), tumor necrosis factor-a (TNF-a), and C-reactive protein (CRP) were quantified using enzyme-linked immunosorbent assay (ELISA) and routine laboratory tests. Logistic regression was applied to identify independent biochemical predictors of CVD, and a risk prediction model was developed and validated using ROC curve analysis.

**Results:**

IL-8, PCT, CRP and age emerged as independent predictors of CVD occurrence in pancreatitis patients (all P&lt; 0.05). The biochemical prediction model demonstrated high accuracy, with an AUC of 0.893 in the training set and 0.978 in the validation set. Sensitivity and specificity exceeded 85% across datasets.

**Conclusions:**

This study highlights the clinical and laboratory significance of inflammatory biomarkers in pancreatitis. The proposed biochemical model provides a reliable tool for predicting cardiovascular risk and may contribute to improved laboratory-guided risk assessment and patient management.

## Abstract (sr)

**Uvod:**

Pacijenti sa pankreatitisom mogu biti izloženi povećanom riziku od kardiovaskularnih bolesti (KVB), ali biohemijski mehanizmi koji leže u osnovi ovog rizika nisu u potpunosti definisani. Inflamatorni biomarkeri mogu pružiti vredne prognostičke informacije.

**Metode:**

Retrospektivno smo analizirali 180 pacijenata sa pankreatitisom (januar 2021 - decembar 2023). Serumski nivoi interleukina-8 (IL-8), prokalcitonina (PCT), faktora nekroze tumora-a (TNF-a) i C-reaktivnog proteina (CRP) kvantifikovani su korišćenjem enzimski povezanog imunosorbentnog testa (ELISA) i rutinskih laboratorijskih testova. Logistička regresija je primenjena za identifikaciju nezavisnih biohemijskih prediktora kardiovaskularnih bolesti (KVB), a model za predviđanje rizika je razvijen i validiran korišćenjem analize ROC krive.

**Rezultati:**

IL-8, PCT, CRP i starost pojavili su se kao nezavisni prediktori pojave KSB kod pacijenata sa pankreatitisom (svi P&lt; 0,05). Bihemijski model predikcije pokazao je visoku tačnost, sa AUC od 0,893 u skupu za obuku i 0,978 u skupu za validaciju. Osetljivost i specifičnost premašile su 85% u svim skupovima podataka.

**Zaključak:**

Ova studija ističe klinički i laboratorijski značaj inflamatornih biomarkera kod pankreatitisa. Predloženi biohemijski model pruža pouzdan alat za predviđanje kardiovaskularnog rizika i može doprineti poboljšanoj laboratorijski vođenoj proceni rizika i lečenju pacijenata.

## Introduction

Pancreatitis is a non-infectious inflammatory condition that is primarily classified into acute pancreatitis and chronic pancreatitis. Acute pancreatitis is characterized by acute injury to the pancreatic tissue, including edema, hemorrhage, and necrosis, caused by various etiological factors. On the other hand, chronic pancreatitis is a progressive inflammatory condition of the pancreas, either localized or diffuse, associated with irreversible damage to both the exocrine and endocrine functions of the pancreas [1] [2]. In China, the main causes of pancreatitis are gallstones leading to impaired pancreatic drainage and long-term alcohol consumption. High-risk individuals include patients with biliary obstruction, gallstones, and chronic alcoholics. The symptoms of pancreatitis vary among individuals, with typical manifestations including acute abdominal pain, nausea, vomiting, fever, and potential signs of acute multi-organ dysfunction and failure, such as hypotension, shock, respiratory distress, oliguria, anuria, upper gastrointestinal bleeding, and sudden death. The treatment approaches for pancreatitis primarily depend on the severity of the disease and specific patient symptoms [3]. Non-surgical treatment options can be employed for patients with mild disease and no signs of infection, while pharmacological or surgical interventions may be necessary for severe cases.

In the case of chronic pancreatitis, the focus is on treating the underlying cause, like managing biliary diseases and abstaining from alcohol [4]. During pancreatitis, pancreatic enzymes are activated, and these enzymes have certain toxic effects. Once activated, pancreatic enzymes can increase vascular permeability, leading to increased fluid extravasation and reduced effective circulating blood volume, which can result in hypotension. Additionally, certain components of pancreatic fluid may directly enter the bloodstream, causing damage to various organs, including the heart muscle. Insufficient effective blood volume also hampers adequate blood supply to the heart, resulting in reduced myocardial perfusion. Prolonged inadequate myocardial perfusion can lead to myocardial injury and heart failure, thereby increasing the risk of cardiovascular disease [5]. Pancreatitis-induced myocardial injury is associated with apoptosis of myocardial cells mediated by inflammatory mediators. These inflammatory mediators play a critical role in the pathological process of pancreatitis and may indirectly or directly cause damage to the heart muscle [6].

Based on these factors, this study aims to investigate a predictive model for the risk of cardiovascular disease occurrence in patients with pancreatitis, specifically focusing on the inflammatory markers in patients with different degrees of pancreatitis, providing clinical reference.

## Materials and methods

### Research objects

180 patients diagnosed with pancreatitis and admitted to our hospital between Jan 2021 and Dec 2023 were included in the study. Inclusion criteria were as follows: (1) Clinical diagnosis of pancreatitis based on the »Diagnosis and Treatment Guidelines for Acute Pancreatitis in China (2021)« [7], including persistent abdominal pain, serum amylase and/or lipase concentrations at least three times higher than the upper limit of normal, and abdominal imaging findings consistent with acute pancreatitis. Patients meeting any two of the above criteria were included. (1) Age ≥ 18 years. (2) Patients experiencing their first episode of pancreatitis. Exclusion criteria were as follows: (1) Patients with underlying diseases involving the brain, liver, kidneys, bone marrow system, or other major organs. (2) Patients who experienced a rapid deterioration of symptoms and developed multiple organ failure within 24 hours of onset. (3) Patients with known allergies to one or more drugs. (4) Pregnant or breastfeeding women. (5) Patients with mental illness or cognitive impairment.

This study has been approved by the Ethics Committee of the hospital. Informed consent was waived for this retrospective study due to the exclusive use of de-identified patient data, which posed no potential harm or impact on patient care. Intelligence (AI)-assisted technologies (such as Large Language Models [LLMs], chatbots, or image creators) are not used in the production of submitted work.

### Research methods

The patients were matched in a 1:4 ratio with data from 180 patients in the same time period to be divided into a validation group (n = 36) and a modeling group (n = 144). The ideal cardiovascular health score was used to assess the risk of developing heart disease in the patients. Based on the scores obtained from the patient questionnaire, the modeling group was divided into a medium-high-risk group (score < 5, n = 50) and a low-risk group (score ≥ 5, n = 94). Inflammatory marker detection: Blood samples were collected from the patients upon admission. Enzyme- linked immunosorbent assay (ELISA) was used according to the instructions provided with the kits to determine the expression levels of inflammatory markers Interleukin-8 (IL-8), Procalcitonin (PCT), and tumor necrosis factor-α (TNF-α) in the patients' blood. Additionally, such traditional inflammatory markers as c-reactive protein (CRP), neutrophils, and six coagulation parameters (prothrombin time (PT), activated partial thromboplastin time (APTT), thrombin time (TT), fibrinogen (Fbg), D-dime, antithrombin III (ATIII), and white blood cells (WBC) were measured through routine blood tests.

### Statistical analysis

The collected experimental data were analyzed with Statistic Package for Social Science (SPSS) 27.0 software (IBM Corp., Armonk, N.Y, USA). For continuous variables, normality tests were conducted, if the data follow normal distribution, continuous variables are presented as mean ± SD and compared using Student's t-test, while non-normally distributed variables are presented as median (inter-quartile range, IQR) and compared using Mann-Whitney U test. Categorical variables are expressed as numbers and percentages, and chi-squared (χ^2^) test was used for comparison between groups. Univariate and binary Logistic regression analyses were conducted to identify factors influencing the risk of heart disease in patients with different degrees of pancreatitis. The predictive value of inflammatory markers for the risk of heart disease was evaluated with receiver operating characteristic (ROC) curves. A significance level of *P*<0.05 was considered statistically significant for all analyses.

## Results

### Comparison of clinical data

Comparison of clinical data between the modeling group and validation group showed no statistically significant differences (*P*>0.05), as shown in Table 1.

**Table 1:** Comparison of Clinical Data. Abbreviations: BMI, body mass index; IL-8, interleukin-8; PCT, procalcitonin; TNF-α, tumor necrosis factor-α; CRP c-reactive protein; PT, prothrombin time; APTT, activated partial thromboplastin time; TT, thrombin time; Fbg, fibrinogen; ATIII, antithrombin III.

General Information		Modeling Group<br>(n = 144)	Validation Group<br>(n=36)	t/χ^2^	P value
Age (years)		52.19±8.16	53.49±7.19	0.874	0.383
Gender	Male	96	21	0.879	0.644
	Female	48	15		
BMI (kg/m^2^)		21.16±3.49	21.33±3.19	0.266	0.791
Hypertension History	Yes	16	6	0.829	0.661
	No	128	30		
Diabetes History	Yes	12	5	1.039	0.595
	No	132	31		
IL-8 (pg/mL)		5.23±1.52	5.65±2.01	1.384	0.168
PCT (ng/mL)		1.34±0.21	1.31±0.11	0.828	0.409
TNF-α (μg/L)		4.61±0.43	4.62±0.22	0.135	0.893
Neutrophils (x10^9^/L)		0.49±0.11	0.51±0.22	0.774	0.440
CRP (mg/L)		5.04±1.03	4.91±0.92	0.691	0.490
White Blood Cells		6.16±1.24	6.22±1.51	0.248	0.804
PT (s)		14.16±2.12	14.55±2.41	0.960	0.338
APTT (s)		41.16±5.13	41.62±5.20	0.480	0.632
TT (s)		17.26±1.11	17.31±1.05	0.244	0.807
Fbg (g/L)		3.09±1.20	3.11±0.98	0.093	0.926
D-dimer (mg/L)		0.83±0.33	0.86±0.28	0.502	0.616
ATIII (μg/mL)		126.72±10.52	127.08±11.05	0.182	0.856
Severity	Mild	99	25	4.416	0.220
	Moderate	20	10		
	Severe	25	11		

### Factors Influencing the risk of developing cardiovascular disease with univariate analysis

Comparison of gender, BMI, hypertension history, diabetes history, TNF-α, neutrophils, leukocytes, pT, APTT, TT, Fbg, D-dimer, and AtIII between the two groups of patients showed no statistically significant differences (*P*>0.05). However, a statistically significant difference (*P*<0.05) was observed in the comparison of IL-8, PCT, CRP age, and disease severity between the two groups. It is suggested that IL-8, PCT, CRP, age and severity may be the risk factors of heart disease. See Table 2 for details.

**Table 2:** Factors Influencing the Risk of Developing Cardiovascular Disease with Univariate Analysis.

General Information		High-Risk Group<br>(n = 50)	Low-Risk Group<br>(n=94)	t/χ^2^	*P* value
Age (years)		54.34±7.22	50.91±7.61	2.621	0.010
Gender	Male	31	65	0.751	0.687
	Female	19	29		
BMI (kg/m^2^)		21.22±2.51	20.31±3.08	1.795	0.075
BMI (kg/m^2^)		7	9	0.647	0.724
Hypertension	Yes	43	85		
	No	4	8	0.011	0.994
Diabetes History	Yes	46	86		
IL-8 (pg/mL)		6.49±1.33	4.61±1.22	8.531	0.000
PCT (ng/mL)		1.43±0.27	1.21±0.20	5.546	0.000
TNF-α (mg/L)		4.62±0.53	4.59±0.45	0.358	0.721
Neutrophils (x10^9^/L)		0.49±0.65	0.48±0.21	0.137	0.891
CRP (mg/L)		5.56±0.65	4.82±0.76	5.840	0.000
White Blood Cells<br>(x10^9^/L)		6.25±1.32	6.12±1.22	0.592	0.555
PT (s)		14.19±2.22	14.12±2.07	0.188	0.851
APTT (s)		41.19±5.01	41.02±5.00	0.194	0.846
APTT (s)		17.32±1.20	17.25±1.23	0.328	0.743
Fbg (g/L)		3.11±0.88	3.08±0.92	0.189	0.850
D-dimer (mg/L)		0.85±0.34	0.82±0.35	0.495	0.622
ATIII (μg/mL)		127.99±11.05	125.73±10.58	1.202	0.231
Severity	Mild	25	74	22.91	<0.001
	Moderate	6	14		
	Severe	19	6		

### Factors influencing the risk of cardiovascular disease with multivariable logistic regression analysis

With IL-8, PCT, CRP age, and disease severity as independent variables, with their actual values assigned, a multivariable logistic regression analysis was conducted to analyze the risk of cardiovascular disease as the dependent variable (high risk = 1, low risk = 0). The results of the multivariable logistic regression analysis indicated that IL-8, PCT, CRP and age were independent factors significantly influencing the risk of cardiovascular disease in patients with varying degrees of pancreatitis (*P*<0.05). See Table 3 for detailed information.

**Table 3:** Factors Influencing the Risk of Cardiovascular Disease with Multivariable Logistic Regression Analysis.

Risk Factor	Value	SE Value	Ward Value	OR Value	95%CI	P Value
IL-8	1.152	0.545	4.469	3.165	1.088-9.211	<0.001
PCT	1.248	0.495	6.361	3.485	1.321-9.195	<0.001
CRP	1.239	0.421	8.661	3.452	1.513-7.878	<0.001
Age	1.261	0.399	9.984	3.528	1.614-7.712	<0.001
Severity	0.381	0.222	2.948	1.464	0.947-2.262	0.053

### Column line graph model construction for predicting the risk of developing cardiovascular disease based on inflammatory markers in patients with varying degrees of pancreatitis

**Figure 1:**
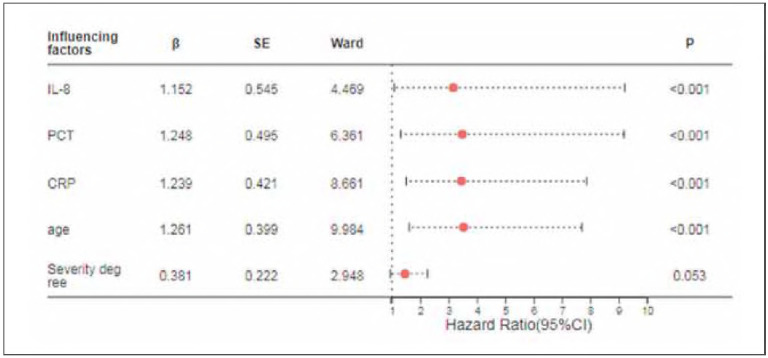
The influence of IL-8, PCT, CRP for CVD occurrence in patients with different degrees of pancreatitis. Abbreviations: IL-8, interleukin-8, PCT, procalcitonin, TNF-α, tumor necrosis factor-α.

**Figure 2:**
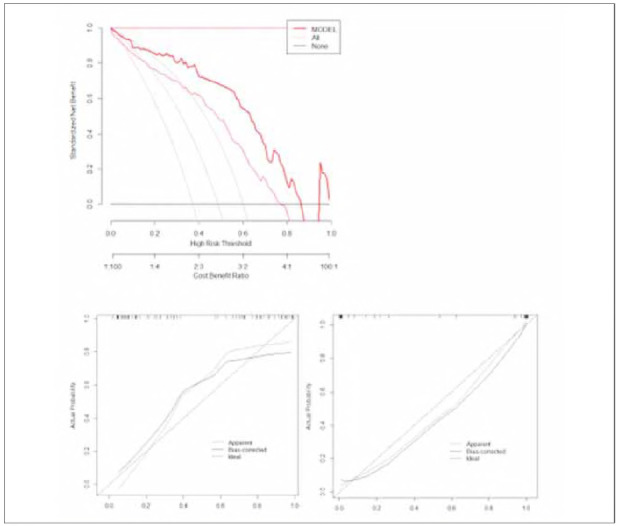
The calibration curve of the model on the training set and the verification set.

Based on the results of the Logistic regression analysis, IL-8, PCT, CRP and age can be used as meaningful influencing factors and can be brought into the construction of the model, as shown in Figure 1. IL-8, PCT, TNF-α, and age were included in the model for predicting the risk of developing cardiovascular disease in patients with varying degrees of pancreatitis, as depicted in Figure 2. The average absolute error of the calibration curve of the model constructed according to the influencing factors is 0.072 in the training set, and 0.043 in the verification set, and the calibration curves of this model in both the training and validation sets show a slope close to 1, indicating good consistency between the predicted and actual risks of cardiovascular disease.

### Value of the model for predicting the risk of cardiovascular disease in the training set and validation set with ROC curve analysis

**Figure 3:**
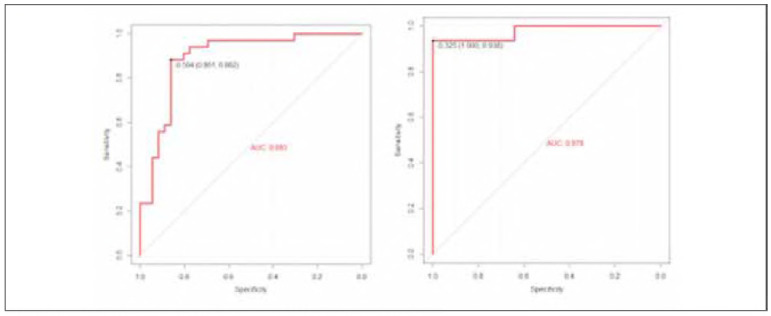
ROC analysis on the training set and the verification set.

ROC showed that in the training set, the model exhibited an area under the curve (AUC) of 0.893, with a standard error of 0.0304 (95% CI: 0.6705-0.9385). The optimal cutoff value was determined to be 0.74, resulting in a sensitivity of 86.1% and specificity of 88.2%, as depicted. In the validation set, the model demonstrated an AUC of 0.978, with a standard error of 0.0325, (95% CI: 0.9725-1.000). The optimal cutoff value was identified as 0.94, yielding a sensitivity of 100.00% and specificity of 93.8%, The results show that the model has good predictive value and is worthy of further use in clinic, as shown in Figure 3.

## Discussion

Mild pancreatitis can lead to manifestations of cardiac dysfunction, such as increased heart rate and cardiac arrhythmias, which are indicative of impaired cardiac function [8]. In the case of severe pancreatitis, the cardiac involvement is usually more severe. The entry of pancreatic fluid into the abdominal cavity can cause vasodilation, inadequate blood volume, and hypotension, thereby affecting the cardiac perfusion function [9]. In this scenario, patients may experience severe cardiac events such as myocardial infarction, cardiogenic shock, ventricular fibrillation, and cardiac arrest, all of which can potentially lead to patient mortality. Additionally, severe pancreatitis can also trigger pericarditis or pericardial effusion, and in some cases, it can result in cardiac tamponade and subsequent patient death [10]. Therefore, there has been increased attention in recent years on studying the risk of cardiovascular disease in patients with pancreatitis.

There were statistically significant differences (*P*<0.05) in IL-8, PCT, CRR age, and severity between the two groups. Further multifactorial analysis revealed that IL-8, PCT, CRR and age were independent influencing factors for the risk of cardiovascular disease in patients with different degrees of pancreatitis (*P*<0.05). The possible reasons for these findings are as follows: IL-8 is an important inflammatory mediator that plays a key role in the pathogenesis of pancreatitis. Elevated levels of IL-8 may lead to myocardial cell damage and apoptosis, thereby increasing the risk of cardiovascular disease. Additionally, IL-8 may promote endothelial cell injury, leading to vascular dysfunction and further exacerbating the occurrence of cardiovascular disease [11]. Furthermore, PCT is an important indicator reflecting the degree of systemic inflammatory response. In patients with pancreatitis, elevated PCT levels may indicate a more severe systemic inflammatory response, which can not only affect the pancreas itself but also cause damage to distant organs like heart[12]. Therefore, increased PCT levels may increase the risk of cardiovascular disease in patients with pancreatitis. CRR as a nonspecific inflammatory marker, is also often elevated in patients with pancreatitis [13]. The elevation of CRP may be associated with myocardial cell damage and inflammatory response, thereby increasing the risk of cardiovascular disease [14]. Lastly, age is an important factor influencing the risk of cardiovascular disease in patients with pancreatitis. With increasing age, organ functions in the body gradually decline. Therefore, elderly patients with pancreatitis are more prone to develop cardiac complications, and their risks of cardiovascular disease accordingly increase [15].

Based on in-depth analysis with Logistic regression, a model was constructed to predict the risk of cardiovascular disease in patients with different degrees of pancreatitis, incorporating four key indicators: IL-8, PCT, TNF-α, and age. During the rigorous model validation process, both in the training and validation sets, the model's calibration curve slope was close to 1, indicating a high consistency between the predicted and actual risks of cardiovascular disease. This demonstrates the excellent predictive ability of the model. Further ROC analysis provided quantitative measures of the model's predictive performance. In the training set, the model achieved a high area under the curve (AUC) for predicting the risk of cardiovascular disease in patients with different degrees of pancreatitis. The small standard error and the 95% confidence interval (CI) also confirmed the reliability of these results. When using the optimal cutoff value as the criterion, the model exhibited satisfactory sensitivity and specificity in identifying true cases of cardiovascular disease risk and excluding low-risk patients. This indicates a high accuracy of the model in clinical application [16]. Similarly, in the validation set, the model demonstrated similar excellent performance. The AUC, standard error, and 95% confidence interval of the predictive curve all indicated the stability and reliability of the model in predicting the risk of cardiovascular disease. Furthermore, the sensitivity and specificity corresponding to the optimal cutoff value in the validation set further validated the effectiveness of the model in practical application. sFinally, decision curve analysis highlighted the clinical utility of the model. By comparing the net benefit at different decision thresholds, it was found that the model had high utility in actual clinical decision-making. It can provide valuable reference information to doctors, assisting them in more accurately assessing the risk of cardiovascular disease in patients with pancreatitis and formulating more scientifically rational treatment plans [17] [18].

Although this study successfully established a model to predict the risk of cardiovascular disease in patients with different degrees of pancreatitis and achieved certain results, there are still some limitations. Firstly, the sample size may be relatively limited. Although this study included a certain number of patient data for model construction and validation, a larger sample size could make the model more stable and reliable [19]. A smaller sample size may limit the model's predictive ability for certain specific or rare cases, affecting its applicability in a broader population. Furthermore, the clinical application of the model still needs further exploration and improvement. Although this study demonstrated that the model has high predictive performance and clinical utility, its application in actual clinical decision-making requires further research and optimization [20].

## Conclusion

In conclusion, this study developed and validated a predictive model for cardiovascular disease occurrence in patients with pancreatitis, with inflammatory biomarkers (IL-8, PCT, and CRP) identified as key independent predictors. By integrating routinely measurable biochemical indicators with clinical data, the model demonstrated excellent sensitivity and specificity, underscoring the diagnostic and prognostic value of inflammatory markers in pancreatitis. These findings highlight the importance of laboratory-based biochemical assessments in risk stratification and may support more precise prevention and management strategies for cardiovascular complications in pancreatitis.

## Dodatak

### Author contributions

Qi Liu - research concept and design, and critical revision of the article; Zaili Yang- collection, analysis and interpretation the data; Dejun Cui, Fei Li, Bo Huang-Investigation; All authors - writing the drafting article and final approval of the article.

### Acknowledgements

The authors express their appreciation to staff in National Institute of Drug Clinical Trials, Guizhou Provincial People's Hospital and Guizhou Medical University, for their technical assistance.

### Funding source

This research was funded by Basic research project of Science and Technology Department of Guizhou Province (Basic of Guizhou Family -ZK Š2022Ć General 368), Marshall Joint Laboratory project of Affiliated Hospital of Guizhou Medical University (N-2021-13) and Guizhou Medical University National Natural Science Foundation cultivation project (21NSFCP16).

### Ethics statement

This study has been approved by the Ethics Committee of Guizhpu Provincial People's Hospital (No.2022394) on 9^th^ March, 2022.

### Consent to participate

Informed consent was waived for this retrospective study due to the exclusive use of de-identified patient data, which posed no potential harm or impact on patient care.

### Consent for publication

Not applicable.

### Data availability statement

All data generated or analyzed in this study are included in the present manuscript.

### Conflict of interest statement

All the authors declare that they have no conflict of interest in this work.
